# Magnetic Lateral Flow Immunoassay for Small Extracellular Vesicles Quantification: Application to Colorectal Cancer Biomarker Detection

**DOI:** 10.3390/s21113756

**Published:** 2021-05-28

**Authors:** Amanda Moyano, Esther Serrano-Pertierra, José María Duque, Virginia Ramos, Estefanía Teruel-Barandiarán, María Teresa Fernández-Sánchez, María Salvador, José Carlos Martínez-García, Luis Sánchez, Luis García-Flórez, Montserrat Rivas, María del Carmen Blanco-López

**Affiliations:** 1 Department of Physical and Analytical Chemistry & Institute of Biotechnology of Asturias, University of Oviedo, c/Julián Clavería 8, 33006 Oviedo, Spain; moyanoamanda@uniovi.es (A.M.); serranoesther@uniovi.es (E.S.-P.); eteruel@ucm.es (E.T.-B.); 2 Hospital Universitario San Agustín, 33401 Avilés, Spain; josemaria.duque@sespa.es (J.M.D.); virginiaramosperez@gmail.com (V.R.); luis.sanchez@sespa.es (L.S.); 3 Department of Medicine, University of Oviedo, 33006 Oviedo, Spain; 4 Department of Biochemistry and Molecular Biology & Institute of Biotechnology of Asturias, University of Oviedo, 33006 Oviedo, Spain; mfernandez@uniovi.es; 5 Department of Physics & IUTA, University of Oviedo, Campus de Viesques, 33204 Gijón, Spain; salvadormaria@uniovi.es (M.S.); jcmg@uniovi.es (J.C.M.-G.); rivas@uniovi.es (M.R.); 6 Hospital Universitario Central de Asturias (HUCA), 33011 Oviedo, Spain; garciafluis@uniovi.es; 7 Department of Surgery and medical-surgical specialties, University of Oviedo, 33006 Oviedo, Spain; 8 Instituto de Investigación Sanitaria del Principado de Asturias (ISPA), 33011 Oviedo, Spain

**Keywords:** colorectal cancer, blood biomarkers, extracellular vesicles, CD147 antigen, magnetic lateral flow immunoassay, inductive sensors

## Abstract

Colorectal cancer (CRC) is the third leading cause of cancer death and the fourth most common cancer in the world. Colonoscopy is the most sensitive test used for detection of CRC; however, their procedure is invasive and expensive for population mass screening. Currently, the fecal occult blood test has been widely used as a screening tool for CRC but displays low specificity. The lack of rapid and simple methods for mass screening makes the early diagnosis and therapy monitoring difficult. Extracellular vesicles (EVs) have emerged as a novel source of biomarkers due to their contents in proteins and miRNAs. Their detection would not require invasive techniques and could be considered as a liquid biopsy. Specifically, it has been demonstrated that the amount of CD147 expressed in circulating EVs is significant higher for CRC cell lines than for normal colon fibroblast cell lines. Moreover, CD147-containing EVs have been used as a biomarker to monitor response to therapy in patients with CRC. Therefore, this antigen could be used as a non-invasive biomarker for the detection and monitoring of CRC in combination with a Point-of-Care platform as, for example, Lateral Flow Immunoassays (LFIAs). Here, we propose the development of a quantitative lateral flow immunoassay test based on the use of magnetic nanoparticles as labels coupled to inductive sensor for the non-invasive detection of CRC by CD147-positive EVs. The results obtained for quantification of CD147 antigen embedded in EVs isolated from plasma sample have demonstrated that this device could be used as a Point-of-Care tool for CRC screening or therapy monitoring thanks to its rapid response and easy operation.

## 1. Introduction

Colorectal cancer (CRC) is the third leading cause of cancer death and the fourth most common cancer in the world, according to GLOBOCAN 2018 data [1]. In Europe, 500,000 new cases of CRC were diagnosed in 2018 (12.8% of all cancers), being the second most frequent cause of cancer death [2]. In Spain, more than 41,000 new cases of CRC are diagnosed each year and more than 15,000 people die every year as a result of this disease [3]. According to SEOM (Sociedad Española de Oncología Médica), CRC was the most frequent malignant tumor diagnosed in Spain in 2019 in both genders (44,937 new cases), being the second in men after prostate cancer and the second in women after breast cancer. Globally, CRC is expected to increase by 60% to more than 2.2 million new cases and 1.1 million deaths by 2030 [4].

Surgery with curative intent is the cornerstone of treatment for CRC and has improved significantly in the last decades. However, the recurrence rate in advanced stages is still high. Early diagnosis is an important point to take into consideration to improve outcomes. The most sensitive test for the detection of colorectal cancer is colonoscopy, which requires an invasive procedure. The complexity and cost of its procedure do not allow colonoscopy as the first option for mass screening most of the population. Currently, the fecal occult blood test has been used widely as a screening test for CRC. However, it displays low specificity. The lack of rapid and simple methods for mass screening makes early diagnosis and therapy monitoring difficult.

Another important factor for an effective CRC screening is the selection and availability of blood biomarkers since their analysis could easily be performed and would lead to a reduction in the overuse of colonoscopy [5]. Extracellular vesicles (EVs) have emerged as a novel source of potential biomarkers, as their number and composition may be altered under different conditions [6,7,8,9,10]. Due to their content in specific proteins, lipids and nucleic acids, they are being considered as a novel source of biomarkers for early detection [11] and prognosis of either primary tumors or metastatic disease, including CRC [12,13,14]. Recently, several studies have been reported in which EVs have been analyzed in the blood of patients with lung [15] or pancreatic [16,17] cancer, obtaining a specificity in the detection of cancer of 75% and 93%, respectively. Regarding CRC, the amount of CD147 expressed in circulating EVs has been found to be significantly higher in CRC cell lines in comparison with normal colon fibroblast cell lines [18]. The specific antigen CD147 or EMMPRIN (extracellular matrix metalloproteinase inducer) is a glycoprotein and a biomarker of interest in cancer [19]. This protein is released by tumour cells and it could be transferred to the surrounding microenvironment and circulation in a soluble form or embedded in circulating EVs [20]. It has been recently described that CD147 is involved in the release of EVs in colon cancer stem cells [21]. Moreover, CD147-containing EVs have been used as a biomarker to monitor response to therapy in patients with CRC [22]. Therefore, this antigen could be used as a non-invasive biomarker for the detection and monitoring of CRC [23].

Despite the potential use as biomarkers, their rapid quantification is a challenge that needs to be addressed. NTA (Nanoparticle Tracking Analysis) and TRPS (Tunable Resistive Pulse Sensing) are two techniques widely used for the characterization and quantification of EVs. However, the long time required for the analysis, the high equipment cost, and the requirement of qualified personnel are the bottlenecks for their use in diagnosis. Thus, their detection would not require invasive techniques and could be considered as a liquid biopsy since they can be isolated from a variety of body fluids (blood, urine and saliva). The development of suitable Point-of-care (POC) devices for the early diagnosis of CRC would be highly advantageous, although the literature on this specific field is still scarce [24,25,26,27].

Our research group has developed the first Lateral flow immunoassays (LFIA) for EVs [28,29]. The platform was applied to EV detection at plasma from chronic fatigue patients [30], and also to the detection of melanoma biomarkers [31]. Lateral flow immunoassays (LFIA) are a powerful POC in vitro test due to their simplicity, rapidity and low-cost [32,33]. Generally, these assays display a negative/positive response that often does not provide enough information to make a diagnosis. Consequently, the development of LFIA with quantitative capacity is a challenge in the search for simple and rapid detection. The replacement of usual reporters such as gold or latex nanoparticles by magnetic nanoparticles (MNPs) could improve the detection [34]. This is possible due to the physical and chemical characteristics of magnetic nanoparticles, which can be detected by means of magnetometric sensors. Magnetic LFIAs have been reported to detect different important analytes in numerous fields [35]. Moreover, magnetic labels not only allow quantification, but they can also be employed for immunomagnetic separation enabling isolation and enrichment of analyte [36]. Limit of detections (LOD) and sensitivity can be enhanced thanks to immunomagnetic separation steps by means of the magnetic tags [37]. Therefore, magnetic nanoparticles could be a suitable label to both improve detection and achieve quantitative LFIA. Recently, we have developed a quantitative magnetic LFIA for EVs detection [38]. For this approach, magnetic LFIAs have been coupled to a sensor developed by the authors to get the quantification for different concentration of isolated EVs from human plasma [38,39,40,41,42].

Here, we propose the development of a magnetic LFIA test combined with an inductive sensor for the non-invasive detection of CRC by CD147 embedded in EVs. This magnetic LFIA could be used as a POC device for screening and monitoring CRC. The device has been applied to human plasma samples from CRC patients.

## 2. Materials and Methods

### 2.1. Chemicals and Apparatus

Anti-tetraspanin antibodies were purchased at Immunostep (Salamanca, Spain): anti-CD9 (clone VJ1/20), anti-CD63 (clone Tea 3/18), and anti-CD147 (clone VJ1/9). Anti-mouse IgG, bovine serum albumin (BSA), 1-ethyl-3-[3-dimethylaminopropyl]-carbodiimide hydrochloride (EDC), N-hydroxysuccinimide (NHS) were provided by Sigma-Aldrich (Madrid, Spain). HEPES was purchased from Fisher Scientific.

Some materials for strips were provided by Millipore (Darmstadt, Germany): glass fibre membrane (GFCP001000) used as sample pad, nitrocellulose membrane (HF07504XSS) and backing cards (HF000MC100). Absorbent pad was purchased from Whatman (Piscataway, NJ, USA). The sample buffer used was 10 mM HEPES pH 7.4 with 0.05% Tween-20 and 1% BSA.

To prepare the detection and control lines, an IsoFlow reagent dispensing system was used. A guillotine Fellowes Gamma (Madrid, Spain) was used to cut the strips. In order to quantify the intensity of the test line using reflectance measurements, a portable strip reader ESE Quant LR3 lateral flow system (Qiagen Inc., Hilden, Germany) was used.

### 2.2. Sample Collection and Isolation of EVs

Patients who participated in the population Colorectal Cancer Screening in Asturias, Spain, and were about to undergo a colonoscopy because of a positive Occult Blood Test in Feces, were recruited. The following exclusion criteria were applied: patients under 18 years; patients who did not give their permission to obtain the sample; patients unable to read, understand explanations or give informed consent; patients affected by any chronic or acute systemic inflammatory process at the time of the examination; patients with active inflammation (ischemic, infectious, chronic inflammatory bowel) in the colon or ileum at the colonoscopy performed; patients with a previous history of neoplasia or affected by extracolonic tumors at the time of the test. Samples were obtained before the endoscopic procedure and after the Consent, which was approved by the Ethics Committee of the Hospital Universitario San Agustín and conformed with the principles outlined in the Declaration of Helsinki. Peripheral venous blood was collected in 10 mL Vacutainer (Becton Dickinson, Franklin Lakes, NJ, USA) tubes with EDTA as an anticoagulant after discarding the first milliliter, and processed within 30 min of collection. Blood was first centrifuged for 30 min at 1550× *g* to remove cells. Aliquots of plasma were stored at −80 °C until use or further centrifuged to isolate extracellular vesicles.

In order to purify the EV, the ExoQuickTM precipitation solution (System Biosciences, Palo Alto, CA, USA) was used following the manufacturer’s instructions. Then, the isolated EV fractions were characterized (concentration and size distribution) using a NanoSight LM10 instrument (Malvern, Worcestershire, UK) and NTA 3.1 software at Nanovex Biotechnologies S.L (Asturias, Spain). To perform the experiments, samples were diluted in 10 mM HEPES 7.4 to achieve a particle concentration about 10^6^–10^7^ EV/mL.

### 2.3. Western Blot Analysis

Isolated EV fractions were homogenised with 1× RIPA buffer (25 mM Tris pH 7.4, 150 mM NaCl, 1% NP-40, 1% NaDeoxycholate, 0.1% SDS). Equal volumes of each sample were mixed with reducing 1× Laemmli-buffer and run on 12% SDS-PAGE gel. Proteins were transferred to PVDF membrane (Amersham; GE Healthcare, Munich, Germany). The membranes were blocked in 5% non-fat dry milk using PBS containing 0.1% Tween20 (PBS-T) and then probed with polyclonal anti-CD63 diluted 1:500 (Santa Cruz Biotech; Santa Cruz, CA, USA) or monoclonal anti-CD147 diluted 1:500 (Immunostep; Salamanca, Spain) overnight at 4 °C. After the washing steps, membranes were incubated with IRDye 800CW goat anti-mouse (LI-COR Biosciences GmbH, Germany; 1:10,000) and then visualised with LI-COR Odyssey^®^ Imaging System (LI-COR Biosciences GmbH, Germany).

### 2.4. Preparation of MNPs-Ab Conjugates

Superparamagnetic magnetite nanoparticles were synthesized following a coprecipitation route [43]. They were coated with a double layer of oleic acid, and then they were bioconjugated to anti-CD63 or anti-CD147 antibodies. Carbodiimide chemistry was used to activate the carboxyl groups of the nanoparticles: 100 μL of EDC (5 mg/mL in MES 50 mM, pH 6.00) and 100 μL of NHS (5 mg/mL in MES 50 mM, pH 6.00) were mixed with 10 μL of nanoparticles. Following 10 min shaking, 50 μL of detection antibody (anti-CD63 or anti-CD147) with a concentration of 1 mg/mL was added, and the mixture was left reacting at room temperature. After 4 h, 100 μL of the blocking solution (1% BSA in PB 10 mM, pH 7.4) was added in order to block the residual carboxyl groups on the particle surfaces. Then, the mixture was separated by a magnet for 10 min. Finally, 300 μL of supernatant was discarded and the pellet was resuspended in 300 μL PB 10 mM, pH 7.4.

### 2.5. Characterization of Conjugates by Dynamic Light Scattering

Size distribution and ζ potential analysis were carried out in order to monitor the conjugation process. The values before and after conjugation process were compared. The equipment used was: Zetasizer Nano ZS ZEN3600 (Malvern Instruments, Malvern, UK) with a solid-state He–Ne laser (633 nm). Measurements were carried out at 25 °C. A reading composed of 30 measurements of the backscattered (173°) intensity was performed. For data processing and analysis, Zetasizer software version 7.03 was used.

### 2.6. Lateral Flow Strips Preparation

Firstly, the nitrocellulose membrane (25 mm wide) was incorporated into a plastic backing card which provides robustness. Then, the detection and control lines of antibodies were dispensed on the membrane using an IsoFlow dispenser at a rate of 0.100 μL/mm. The test line provides information of the analysis following a sandwich format and the control line validates the test indicating that the liquid sample has flowed adequately along the strip. The concentration and antibodies dispensed in each case were: 1 mg/mL of anti-CD9 (clone VJ1/20) and 1 mg/mL of anti-IgG for the test and the control lines, respectively. Then, the nitrocellulose membrane was dried for 20 min at 37 °C to ensure the immobilization of the antibodies. The sample and the absorbent pads were assembled onto a backing card overlapping the components by 2 mm. Finally, the complete card was cut into individual 5 mm-wide dipstick strips.

### 2.7. Optical Quantification

Reflectance measurements were performed to quantify the color intensity of the test line. For this, a portable strip reader ESE-Quant LR3 lateral flow system (Qiagen Inc., Germany) was used. This equipment contains two channels (LED excitation and photodiode detection) in order to scan the strip by illuminating it with a light beam. Then, a confocal detector measures the attenuation from the surface of the strip, registering the signal and converts it into an electrical signal, which is related to the amount of analyte at the test lines. The unit provided by this device is mm·mV, resulting from integrating the electrical signal (mV) across the width of the test line (mm).

### 2.8. Magnetic Quantification

A quantitative magnetic detection of the test lines was carried out using a home-made inductive sensor specially adapted for scanning LFIA strips [44].

The sensing head is a planar inductor printed on a rigid insulating substrate. To monitor continuously the magnitude and phase of the coil impedance, a precision impedance analyzer (Agilent 4294A) is employed using 16048G test leads and 500-mV/20-MHz excitation voltage. The LFIA strips were scanned laterally over the sensing head using a micro-positioner. During the scanning, an impedance increase is induced by the magnetic permeability change produced by the magnetic particles. The impedance peak is integrated over the position to account for all the particles in the line, no matter how they are distributed.

The sensing coil has the twofold role of exciting and detecting the particles. As there is no need of additional external magnetic field to magnetize the particles, the system complexity is kept minimal.

The units provided by this sensor are µΩ·mm coming from the cumulative integral of the impedance (µΩ) across the width of the test line (mm). It has been demonstrated that the impedance change is directly proportional to the number of superparamagnetic nanoparticles at the test line, which enables to determine the analyte concentration [38,41,42].

## 3. Results and Discussion

### 3.1. Detection of EVs Using LFIA

**Figure 1:**
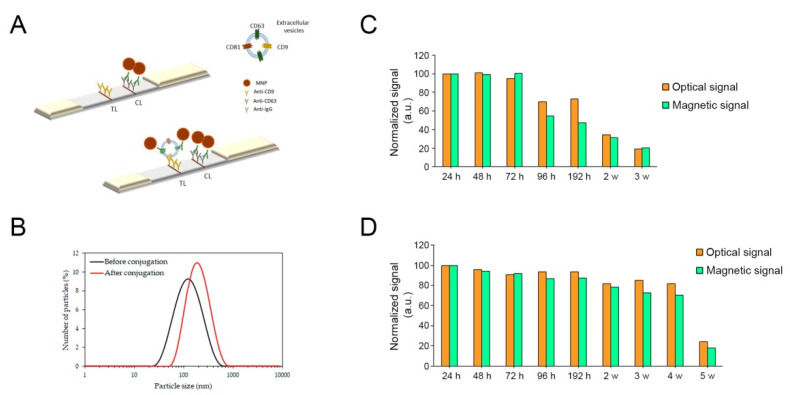
(**A**) Simplified schematic illustration of the LFIA. Specific antibodies against tetraspanins (test, TL) and anti-mouse immunoglobulin antibodies (control, CL) are immobilized on the membrane. Blank test (upper graph): when there are no extracellular vesicles in the sample, the test line does not appear. Sample test (bottom graph): when there are extracellular vesicles in the sample, they are detected by the detection of the complex MNPs-Ab displaying the test line. (**B**) Hydrodynamic size distribution profiles of superparamagnetic iron oxide nanoparticles before (solid black line) and after the conjugation with anti CD63 antibody concentration of 1 mg/mL (red). (**C**) MNPs-Ab conjugates stability measurements at room temperature for 24, 48, 72, 96, 192 hours (h) h, 2 and 3 weeks (w). (**D**) MNPs-Ab conjugates stability measurements at 4 °C for 24, 48, 72, 96, 192 h, 2, 3, 4 and 5 weeks.

The immunoassay developed to detect extracellular vesicles is based on a sandwich format using two antibodies against tetraspanins CD9 and CD63. Both tetraspanins are membrane proteins contained in most EVs. Therefore, they can be considered generic biomarkers of EVs. They were chosen according to previous results obtained in our research group [28,29], and optimized for the samples in this work. The anti-CD9 antibody and anti-CD63 antibody were used for capture and detection, respectively. EVs samples were diluted in running buffer to achieve a particle concentration ranging from 8–94.2 × 10^7^ EVs/µL. For LFIA, different aliquots of MNPs-Ab conjugates and EVs were added in a microtube. The sample pad of the dipstick was immersed in the tube, so the sample flowed by capillarity and MNPs-Ab-EVs complexes are retained at the test line by molecular recognition at anti-CD9 antibody immobilized at the test line (Figure 1A). MNPs-Ab-EVs complexes were added in excess to ensure that there are enough MNPs-Ab conjugates to be retained at the control line using the anti-mouse immunoglobulin antibodies, which validates the test. For the blank, the liquid sample does not contain EVs, and therefore MNP-Ab were retained only at the control line (MNPs-Ab-EVs complexes were not formed due to the lack of analyte). Figure 1A schematically represents the immunoassay on the strip.

### 3.2. Characterization of MNPs-Ab Conjugates

DLS measurements were used to follow the conjugation process by comparing the hydrodynamic diameter of the bare nanoparticles and the MNPs-Ab conjugates. Figure 1B shows the increase in the average diameter after bioconjugation: from 105.5 nm (PDI 0.2) to 188.4 nm (PDI 0.2), respectively. This result (an increment of 83 nm approximately) confirms the success of this step. It also indicates that the carbodiimide chemistry protocol employed was suitable to activate the surface groups and the amount of detection antibody used during the conjugation process was effectively optimized.

ζ potential was used to assess the stability of the bare nanoparticles and the MNPs-Ab. The values of ζ potential obtained were −53.7 and −37.5 mV for bare nanoparticles and MNPs-Ab, respectively. These results indicate that the nanoparticles were very stable in solution due to the electrostatic repulsions between the carboxyl groups provided by oleic acid coating on their surface. The ζ potential value of MNPs-Ab was still high enough to indicate that the conjugates are stable in solution.

### 3.3. Stability of MNPs-Ab Conjugates

The signal stability over time of MNP-Ab was studied at room temperature and 4 °C. For this purpose, the signal was evaluated following the same protocol explained before. The strips have been tested at different times (24, 48, 72, 96, 192 h, 2, 3, 4 and 5 weeks after bioconjugation).

Figure 1C shows the optical and magnetic signals for the different times at room temperature. The optical signal was stable for 72 h, maintaining 100% of the signal. After 72 h, 30% of the signal intensity was lost. After 2 weeks, the optical signal decreased 66%, and after 3 weeks, by 81%. These results indicate that the test should be carried out within 72 h when MNPs-CD63 conjugates are stored at room temperature in order to get the highest efficiency. This decrease could be related to the antibody stability at room temperature, which would affect the conjugates used as labels in the tests. The tendency for the magnetic graph is similar to that observed with the optical readings. The signal was stable for 72 h and then decreased with time.

The signal loss has been studied at 4 °C to investigate whether the conjugated antibody was more stable at this temperature. As Figure 1D shows, the optical and magnetic signals were stable within 192 h, and after 2 weeks, a loss of 20% was observed. After 5 weeks, only 20% of both signals was detected, registering a loss of 80%. It is worth noticing that the MNPs-CD63 conjugates are more stable at 4 °C than at room temperature, enabling to improve their highest stability more than one week. The results indicate that the conjugates have to be kept in the fridge in order to avoid their loss of effectiveness in the signals provided in LFIA. This agrees with the recommendations for anti-CD63 storage, which should be kept at 4 °C according to the manufacturer’s instructions.

Taking into account the MNPs-Ab conjugate stability at both temperatures, the tests should be better carried out within 72 h if the conjugates are kept at room temperature or within two weeks if they are kept at 4 °C.

### 3.4. Extracellular Vesicles Characterization

EV fractions used to develop the magnetic LFIA were isolated from human plasma using a commercial precipitation reagent (ExoQuickTM, System Biosciences, Palo Alto, CA, USA). This has demonstrated to be suitable for EVs isolation and subsequent analysis by LFIA [29,30], even better than other ones [45].

**Figure 2:**
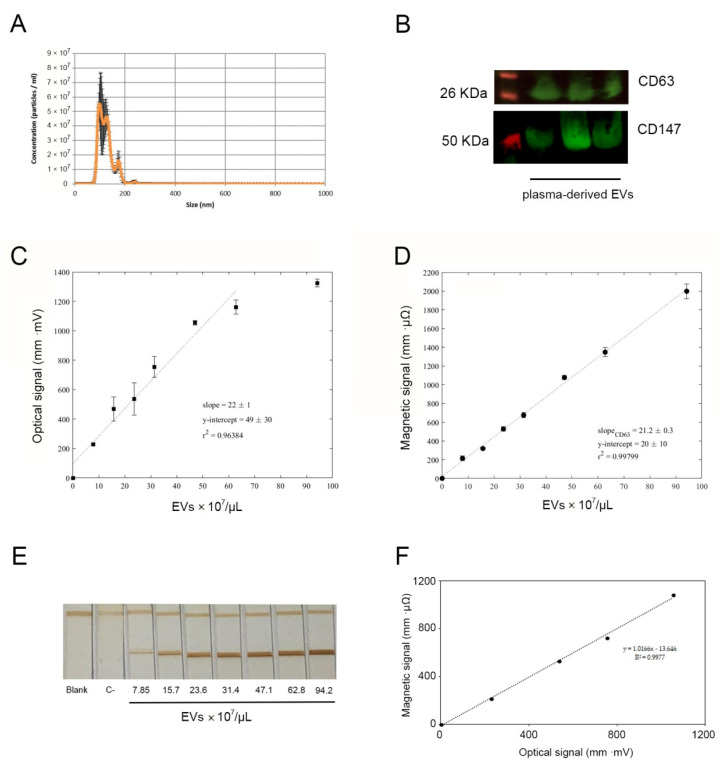
(**A**) Representative graph depicting the concentration and the hydrodynamic size distribution profiles of isolated EV, measured by NTA. (**B**) Western Blot analysis for detection of CD63 and CD147 in EVs fractions. Optical (**C**) and magnetic (**D**) calibration curve obtained in a range of EV concentrations, using anti-CD9 as capture antibody and MNP-anti-CD63 as detection probe. The measurements were obtained in triplicate; the graph shows the mean +/- standard deviation (SD) (**E**). Representative immunostrip tests obtained in the calibration experiments. (**F**) Correlation analysis between the measured optical and magnetic signals of LFIA strips for CD63 detection from 0 to 62.8 × 10^7^ EVs/µL.

EVs purified from plasma were characterized by NTA to determine the size distribution and concentration. Isolated EV fractions were further analysed to determine their concentration and size distribution by NTA. The number of EVs ranged from 3.12 × 10^13^ to 4.38 × 10^13^ particles/mL. Figure 2A shows the average size of the EVs from nanoparticle tracking analysis (NTA) which was 129 nm (range from 118–136). According to the International Society for Extracellular Vesicles, these biological vesicles could be considered “small EVs,” since their size is <200 nm [46]. The presence of the commonly used marker CD63 in EV fractions was determined by Western blot; levels of CD147, the biomarker of interest used to further implement the magnetic LFIA, were also evaluated by Western blot (Figure 2B).

### 3.5. Calibration

In order to calibrate the system, different concentrations of EVs purified from human plasma in the range of 7.85–94.2 × 10^7^ EVs/µL were used. The protocol to perform the tests was explained in Section 3.1 using anti-CD63 as the detection antibody. Subsequently, the optical and magnetic signals were analyzed to study the relationship between the analytical signals and the concentration.

Figure 2E shows the strips performed with different concentrations of EVs isolated from plasma of CRC patients. As corresponds to a sandwich format immunoassay, the signal increases with the concentration. This can be confirmed by observing the intensity of the test lines by naked eye. Figure 2C,D show the quantitative analysis for optical and magnetic sensor, respectively.

Table 1 shows the comparison of the parameters obtained from the fit of the experimental data (linear ranges, regression parameters, and LODs) from both the optical and inductive sensors. The optical reader is based on the reflectance measured from the nitrocellulose strip. In this case, only the nanoparticles at the strip’s top layer can be detected. Due to this feature, the saturation concentration should be reached before than with other methods based on detection of nanoparticles within full thickness of the membrane. This is the case of the inductive sensor employed also in this work to quantify the test line, which provides a larger linear range and a better coefficient of determination as given in Table 1. The LOD was calculated following the 3$\sigma_{b}/m$ criterium, where m is the slope and $\sigma_{b}$ the standard deviation of the y-intercept. The value achieved with the inductive sensor is better than that obtained by optical reader. Moreover, the LOD could be improved by using the potentiality of magnetic nanoparticles to preconcentrate. The error bars obtained for the inductive sensor are smaller showing a better variability compared to the optical sensor.

Moreover, both measuring principles are correlated (Figure 2F), since a good linear correlation was found (R^2^ = 0.9977). This validates the experimental in-house inductive sensor and demonstrates the dual-signal LFIA.

### 3.6. Stability of Optical and Magnetic Signals over Time

**Figure 3:**
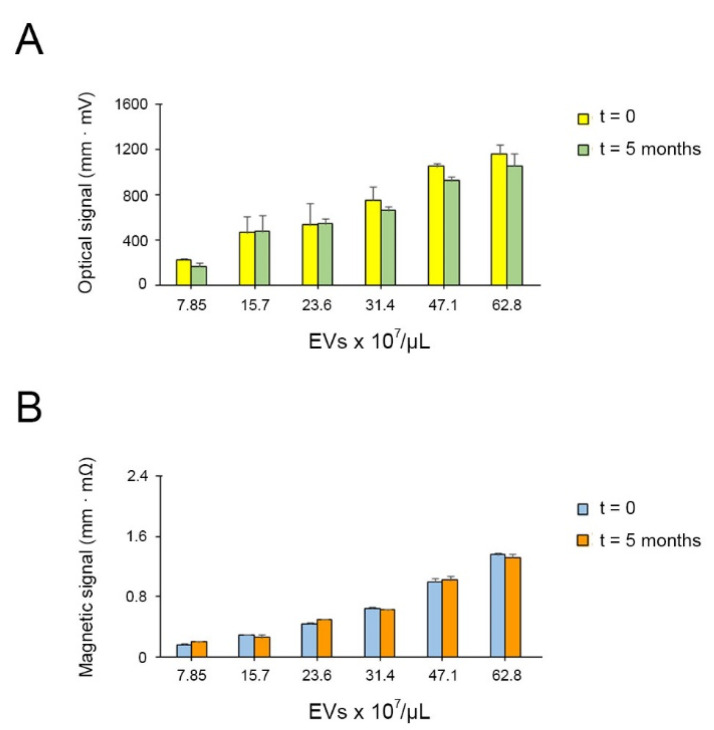
(**A**) Optical signal stability measurements over time. (**B**) Magnetic signal stability measurements over time.

In order to evaluate the signal stability and aging, optical and magnetic signals of the strips used for calibrations have been measured five months after the test was run. The signals were compared with those measured immediately. Figure 3A,B show that the stability was maintained over time for both cases, with no significant differences between them. As a result, it was confirmed that strips could be checked for the optical and magnetic reading at least five months after running the test.

### 3.7. Development of a Magnetic LFIA Targeting CD147

After testing the stability of the conjugates and the signal intensities over time, the LFIA was adapted for the detection of the specific CD147 biomarker at EVs. The number of CD147 + EVs has been reported to be altered in colorectal cancer [18,22,23]. For this purpose, the anti-CD63 previously used as detection antibody was replaced by anti-CD147. Two different clones of the antibody were tested previously to select the most suitable for functionalization of the MNPs and subsequent detection of EVs expressing CD147 (data not shown). Blood samples from patients with CRC were collected and EVs were isolated following the procedures described previously. The optical reader and inductive sensor were used to get quantitative measurements of the test lines.

**Figure 4:**
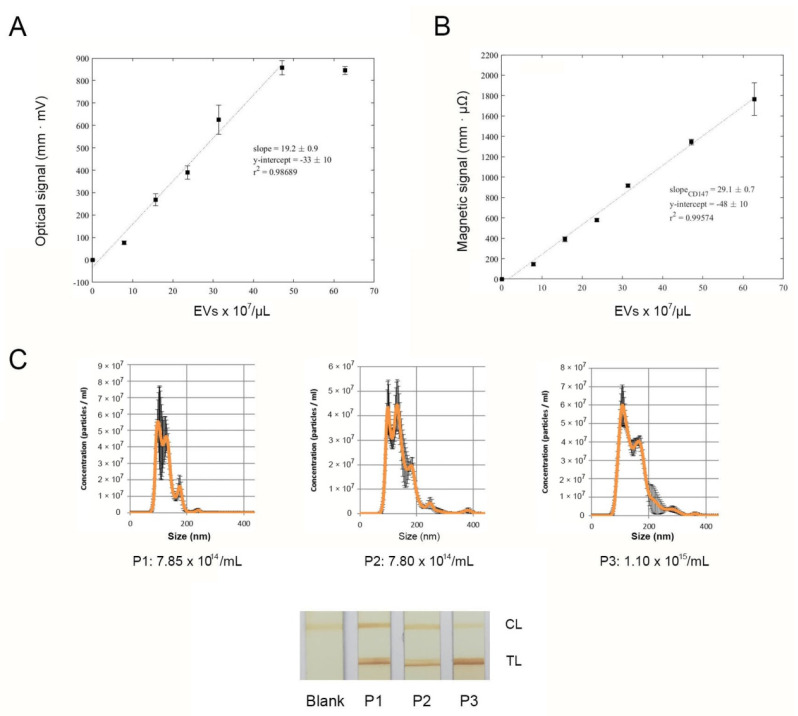
Optical (**A**) and magnetic (**B**) signal intensities obtained in a range of EV concentrations, using anti-CD9 as capture antibody and MNP-anti-CD147 as detection probe. (**C**) Analysis of isolated EVs from three CRC patients. Size and concentration were determined by NTA and CD147 + EVs were detected by LFIA; tests were run in triplicates and the representative immunostrip tests obtained are shown.

First, EVs fractions from the same sample and ranging from 0 to 62.8 × 10^7^ EVs/µL were analyzed. Test line intensities were read and averaged for each concentration using the optical reader and the inductive sensor (Figure 4A,B, respectively). The signal intensities increased with increasing concentrations of EVs and showed good linear relationships in both cases. Thus, the system adapted for targeting CD147 offered a good performance.

Next, three different plasma-derived EV samples from CRC patients were characterized in terms of size and concentration by NTA, and the LFIA analysis was performed upon isolation from the same starting volumes (Figure 4C). Differences in the test line values were observed, even for samples with similar concentrations of EV (P1 and P2). These preliminary results indicate that the immunochromatographic strips prepared in this study enable the analysis of CD147 + EVs, recently described as potential biomarkers in colorectal cancer.

This approach allows its use as POC test to carry out screening of CRC patients, either with optical or magnetic detection, providing, therefore, a decentralized platform that does not need expensive instrument, laborious protocols, or skilled personnel. The total time from isolation of EVs and results was 2 h. However, further work is required to find a correlation between the signal obtained by LFIA and the clinical significance.

## 4. Conclusions

This work shows the quantification of total EVs by means of magnetic lateral flow immunoassays. For total EVs content, the detection of the tetraspanin CD63 was used. Then, a LFIA was designed to detect EVs expressing the signaling protein CD147, a potential CRC biomarker. In both cases, magnetic nanoparticles with a coating double layer of oleic acid were used as reporter labels. 

The LODs achieved for total EVs content determination were 3 × 10^7^ and 1 × 10^7^ EVs/µL for optical and magnetic quantification, respectively. The MNPs-Ab conjugates were stable at least for 72 h and two weeks after conjugation process when the conjugates were kept at room temperature and 4 °C, respectively. The experimental inductive sensor was validated by the correlation with the commercial optical reader based on reflectance measurements. The optical and magnetic signals displayed by MNPs at the test lines were stable over time at least five months. With these immunochromatographic tests, it was possible to analyze specific EVs biomarkers in 2 h using a simple and user-friendly device.

The results obtained in this study suggest that the device proposed could be useful for CRC screening or monitoring of therapies in patient samples. They are rapid and user-friendly, so they could be considered as POC devices, hence, overcoming the drawback of other techniques such as colonoscopy in selected patients.

## Figures and Tables

**Table 1:** Analytical Figures of merit for quantification of EVs by optical and magnetic signals of magnetic nanoparticles.

Quantification	Lineal Range	Slope (Signal Units/(EVs/µL))	Intercept (Signal Units)	LOD (EVs/µL)	Regression Coefficient R^2^
Optical	0–62.8 EVs × 10^7^/µL	22 ± 1	49 ± 30	3 × 10^7^	0.96384
Magnetic	0–94.2 EVs × 10^7^/µL	21.2 ± 0.3	20 ± 10	1 × 10^7^	0.99799
